# Supplementary material for: C. elegans models of Alternating Hemiplegia of Childhood have dominant neuromuscular junction defects
Source: bioRxiv. 2026 Apr 26:2026.04.22.720250. Preprint. [Version 1] doi: 10.64898/2026.04.22.720250 (PMC13131465; doi:10.64898/2026.04.22.720250)
Supplement: Supplement 2 [file NIHPP2026.04.22.720250v1-supplement-2.pdf]

bioRxiv preprint doi: https://doi.org/10.64898/2026.04.22.720250; this version posted April 26, 2026. The copyright holder for this preprint (which was not certified by peer review) is the author/funder, who has granted bioRxiv a license to display the preprint in perpetuity. It is made available under aCC-BY 4.0 International license.

Supplementary Figures PDF

```
ATP1A3   795    ITILCIDLGTDMVPAISLAYEAAESDIMKRQPRNPRTDKLVNERLISMAYGQIGMIQALG  854
                +TILCIDLGTDMVPAISLAYE AESDIMKRQPR+P  DKLVNERLIS+AYGQIGMIQA
EAT-6    778    VTILCIDLGTDMVPAISLAYEEAESDIMKRQPRDPIRDKLVNERLISLAYGQIGMIQASA  837

ATP1A3   855    GFFSYFVILAENGFLPGNLVGIRLNWDDRTVNDLEDSYGQQWTYEQRKVVEFTCHTAFFV  914
                GFF+YF I+A+NGF+P +L  +R  WD R  N++ DSYGQ+WTY  RK++E+TC TA+FV
EAT-6    838    GFFTYFWIMADNGFMPWDLYQLRAQWDSRAYNNVLDSYGQEWTYANRKILEYTCQTAYFV  897

ATP1A3   915    SIVVVQWADLIICKTRRNSVFQQGMKNKILIFGLFEETALAAFLSYCPGMDVALRMYPLK  974
                SIVVVQWADLII KTRRNS+ QQGM N  L FGL  ETALA F+ YCPG+D  LRMY L+
EAT-6    898    SIVVVQWADLIISKTRRNSLVQQGMSNWTLNFGLVFETALAWFMCYCPGLDNGLRMYGLR  957
```

Supplemental Figure 1: Partial alignment of Human ATP1A3 and *C. elegans* EAT-6
Conserved amino acid residues shown between the two protein sequences for human ATP1A3 (NP_689509.1) and *C. elegans EAT-6* (B0365.3); similar amino acids indicated with "+". Bold indicates transmembrane domains 6 through 9. Highlighted residues indicate location of patient missense mutations inserted into endogenous *eat-6* via CRISPR/Cas9 to create *C. elegans* AHC models for D801N (red), E815K (blue), L839P (green), or G947R (purple).

bioRxiv preprint doi: https://doi.org/10.64898/2026.04.22.720250; this version posted April 26, 2026. The copyright holder for this preprint (which was not certified by peer review) is the author/funder, who has granted bioRxiv a license to display the preprint in perpetuity. It is made available under aCC-BY 4.0 International license.

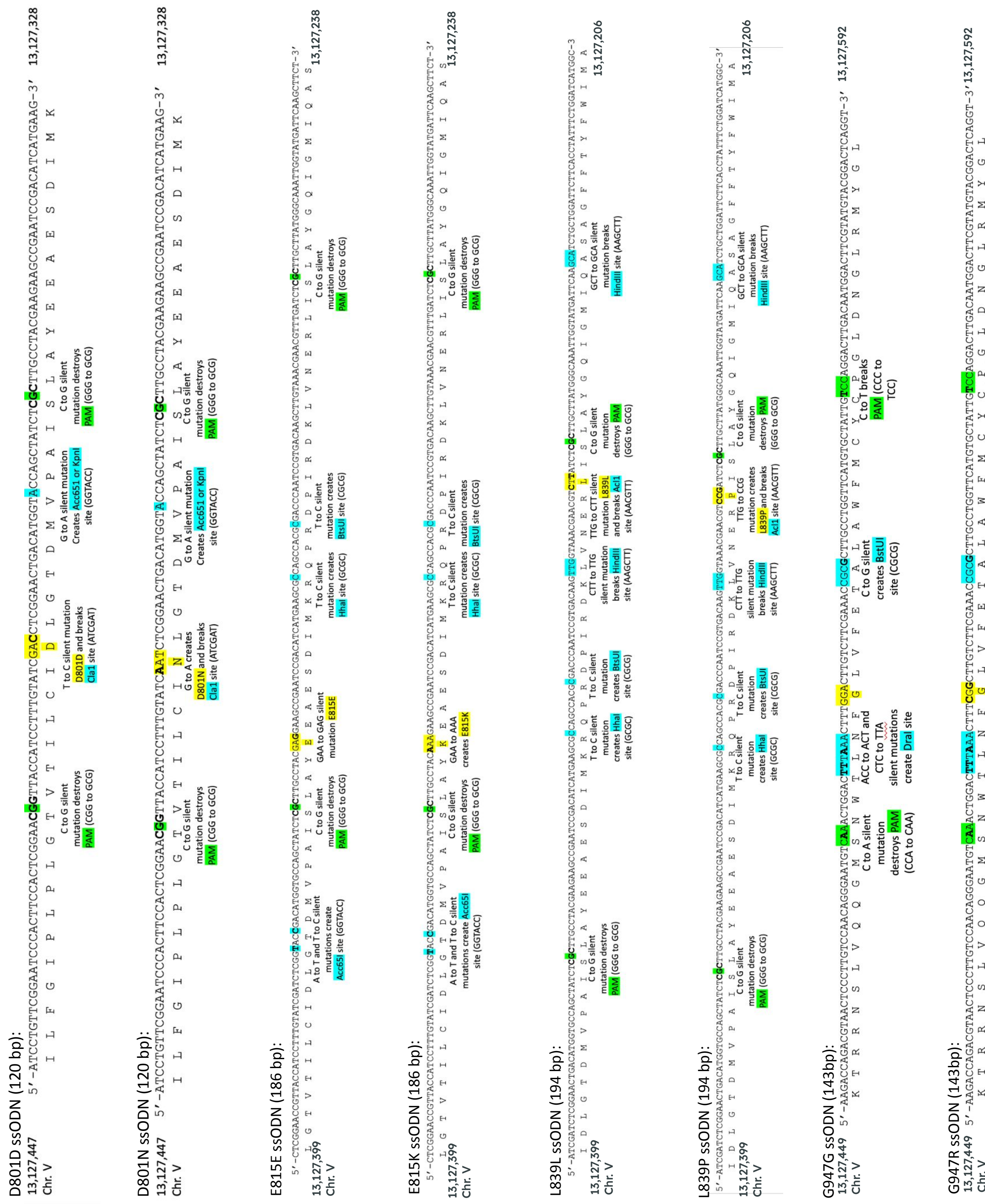

Supplemental Figure 2: ssODNs used for generation of each AHC model and CRISPR control strain
DNA sequence of each ssODN is displayed above its translated amino acid sequence. Location on chromosome is noted. Each ssODN contains 30bp of unedited flanking homology arms, silent mutations to destroy sgRNA PAM sites (green), AHC patient missense mutation for AHC model strains and CRISPR control silent mutations for control strains (yellow), and silent mutations to introduce or remove restriction sites for PCR-based genotyping (blue).

bioRxiv preprint doi: https://doi.org/10.64898/2026.04.22.720250; this version posted April 26, 2026. The copyright holder for this preprint (which was not certified by peer review) is the author/funder, who has granted bioRxiv a license to display the preprint in perpetuity. It is made available under aCC-BY 4.0 International license.

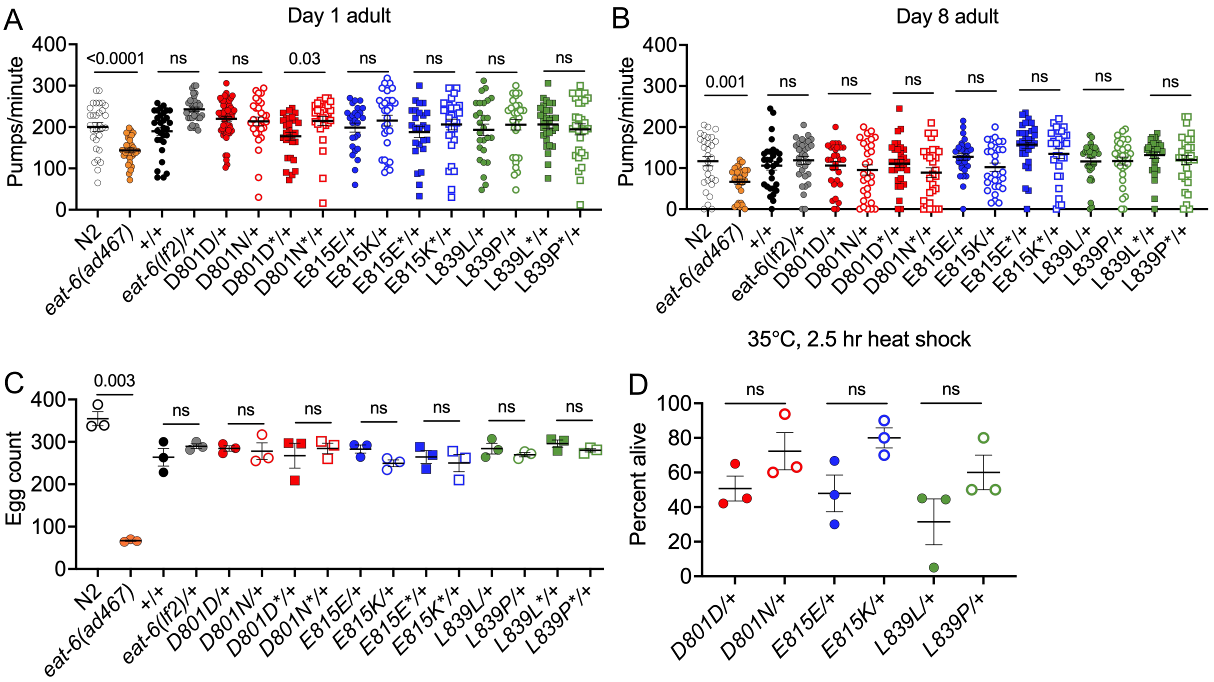

Supplemental Figure 3: Pharyngeal pumping, egg-laying, and stress-response of AHC model animals
Pharyngeal pumps per minute in day 1 adult (A) and day 8 adult (B) animals. Data accumulated from three trials, n=26-30 animals per genotype. N2, *eat-6(ad467), +/+, eat-6(lf2)/+* tested in a one-way ANOVA with Šídák's multiple comparisons test between shown pairs. One-way ANOVA of +/+, the heterozygous primary and replicate CRISPR control strains, and heterozygous primary and replicate AHC model strains tested in with Šídák's multiple comparisons test between each CRISPR control and AHC mutant pair (shown on graph) and +/+ and each CRISPR control (not shown on graph P>0.05). For day 1 adults, +/+, D801D/+, D801D*/+, D801N/+, D801N*/+ were assayed separately from the other genotypes, but data is combined for presentation in A. Statistics were performed only on animals tested in parallel. (C) Number of eggs laid by 8 adults in 6 hours. Three biological replicates. One-way ANOVAs between +/+, two replicate AHC model strains, and their two replicate CRISPR control strains with Šídák's multiple comparisons test between +/+ and each CRISPR control strain, and between each AHC mutant and its respective CRISPR control. This analysis was performed for each AHC allele set. Comparisons between +/+ and CRISPR control strains not shown on graph (all P>0.05 except +/+ vs E815E*/+ P=0.0020). Unpaired t-tests with Welch's correction between N2 and *eat-6(ad467)* and between +/+ and *eat-6(lf2)/+*. (D) Percent of heterozygous adult animals alive 60 hours after a 2.5 hour, 35°C heat-shock. n=18-20 animals per genotype per trial. Three biological replicates. One-Way ANOVA with Šídák's multiple comparisons test between shown pairs. In all panels, One "+" in heterozygous animals represents the *nT1* balancer, error bars indicate mean +/- SEM, ns P>0.05.

bioRxiv preprint doi: https://doi.org/10.64898/2026.04.22.720250; this version posted April 26, 2026. The copyright holder for this preprint (which was not certified by peer review) is the author/funder, who has granted bioRxiv a license to display the preprint in perpetuity. It is made available under aCC-BY 4.0 International license.

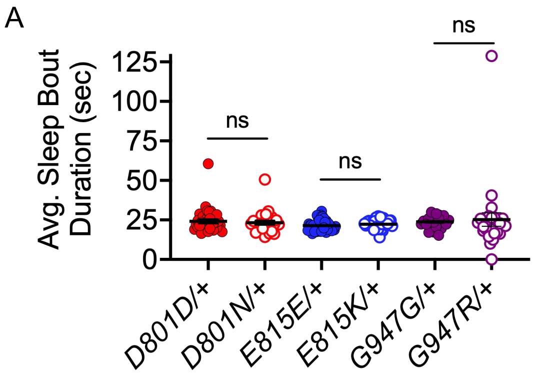

Supplemental Figure 4: Average sleep bout duration in heterozygous AHC model animals
Average sleep bout duration for D801N/+, E815K/+, and G947R/+ model animals and corresponding CRISPR control strains using Welch's unpaired t-test. n= 34-21 animals per genotype. "+" chromosome in D801D/+, D801N/+, E815E/+, and E815K/+ animals is the *nT1* balancer. "+" chromosome in G947G/+ and G947R/+ animals is the *tmC12* balancer. Error bars represent mean +/- SEM.

bioRxiv preprint doi: https://doi.org/10.64898/2026.04.22.720250; this version posted April 26, 2026. The copyright holder for this preprint (which was not certified by peer review) is the author/funder, who has granted bioRxiv a license to display the preprint in perpetuity. It is made available under aCC-BY 4.0 International license.

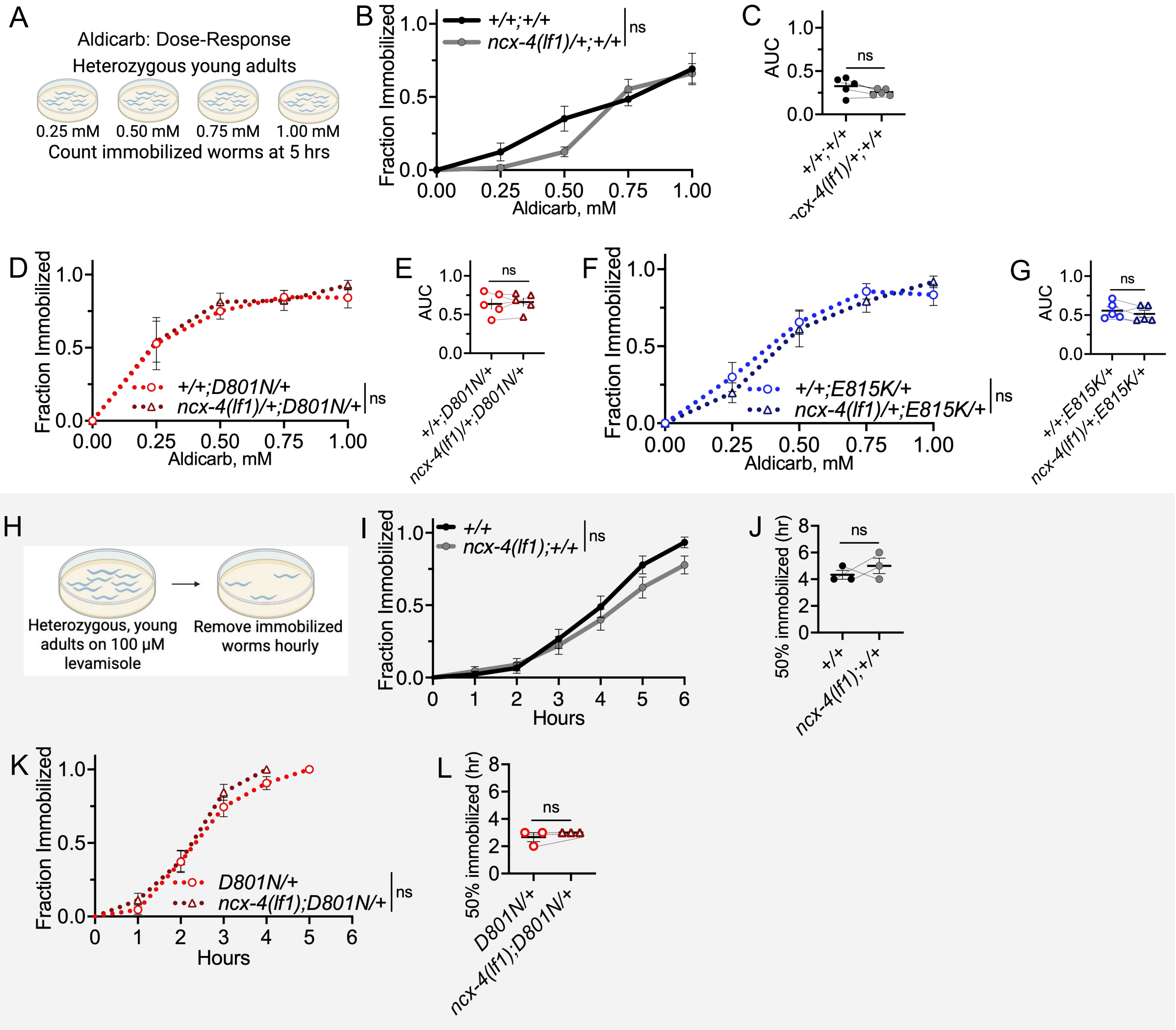

Supplemental Figure 5: Heterozygous loss of ncx-4 did not alter AHC model aldicarb defects, and homozygous loss of ncx-4 did not suppress D801N/+ levamisole defects

(A) Aldicarb dose-response assay. The number of young-adult animals immobilized on 0, 0.25, 0.5, 0.75, and 1 mM aldicarb was recorded at five hours. (B) Dose-response curve for *ncx-4(tm5106)/+;+/+* and +/+ animals. One "+" chromosome is the *tmC18* balancer, and one "+" chromosome is the *tmC12* balancer in all strains used in B-G. Two-way ANOVA. (C) Area under the curve (AUC) from each trial in B, Paired t-test. (D) Dose response curves for D801N/+ animals with and without one copy of *ncx-4(tm5106).* Two-way ANOVA. (E) AUC from each trial in D. Paired t-test. (F) Dose response curves for E815K/+ animals with and without one copy of *ncx-4(tm5106).* Two-way ANOVA. (g) AUC from each trial in F. Paired t-test. For all two-way ANOVA analysis, the displayed P value results from genotype as the source of variation. Data for each genotype results from five biological replicates. For each trial, n=13-15 animals per genotype. (H) Levamisole time-response assay. The number of heterozygous, young-adult animals immobilized on 100 uM levamisole was recorded hourly for six hours. (I) Immobilization curve for *ncx-4(tm5106);+/+* and +/+ animals. In all strains in I-L, one "+" chromosome is the *tmC12* balancer. Mantel-Cox log-rank analysis. (J) Median immobilization time for each trial in I. Paired t-test. (K) Immobilization curve for D801N/+ animals with and without one copy of *ncx-4(tm5106).* Mantel-Cox log-rank test. (L) Median immobilization time for each trial in K. Paired t-test. Data for each genotype is from three biological replicates. For each trial, n=13-15 animals per genotype. All error bars indicate mean +/- SEM, ns indicates P>0.05.

bioRxiv preprint doi: https://doi.org/10.64898/2026.04.22.720250; this version posted April 26, 2026. The copyright holder for this preprint (which was not certified by peer review) is the author/funder, who has granted bioRxiv a license to display the preprint in perpetuity. It is made available under aCC-BY 4.0 International license.

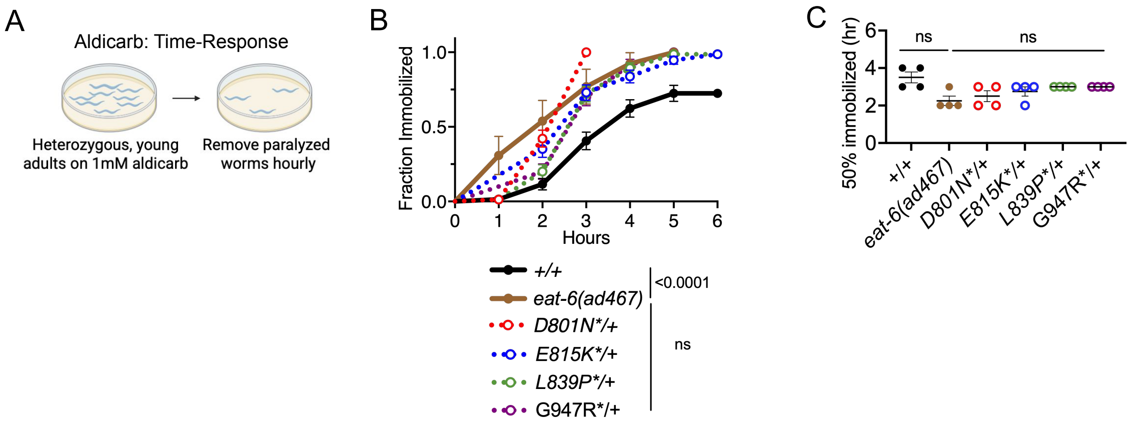

Figure S6: Dominant NMJ defects in AHC model animals are similar in magnitude to *eat-6(ad467)* animals: aldicarb time-response

(A) Aldicarb time-response assay. The number of heterozygous, young-adult animals immobilized on 1 mM aldicarb was recorded hourly. (B) Immobilization curves for wild-type +/+, homozygous *eat-6(ad467)* partial loss-of-function, and heterozygous AHC model D801N/+, E815K/+, and L839P/+ animals. Mantel-Cox log-rank analysis performed between +/+ and *eat-6(ad467)* animals, and between *eat-6(ad467)* and each AHC model strain. (C) Median immobilization time for each trial in B. Paired t-test between between +/+ and *eat-6(ad467)* animals, and between *eat-6(ad467)* and each AHC model strain. Each genotype tested in four biological replicates. For each trial, n=10-22 animals per genotype. Error bars indicate mean +/- SEM. ns indicates P>0.05. In all strains except *eat-6(ad467),* one "+" represents the *tmC12* balancer. Data for +/+, D801N*/+, E815K*/+, L839P*/+, and G947R*/+ is re-used from Figure 4. Note that G947R*/+ model animals animals were assessed in separate trials but are presented together for visual purposes to compare relative magnitude of aldicarb hypersensitivity.
